# Comparison of Self-Reported and Capacity-Based Measures of Mobility in Community-Dwelling Older Adults in Nigeria: The Mediating Role of Age, Cognitive Status, and Chronic Conditions

**DOI:** 10.1093/geroni/igae026

**Published:** 2024-02-29

**Authors:** Michael E Kalu, Daniel Rayner, Ernest C Nwachukwu, Michael C Ibekaku, Miracle Ndukaku, Uduonu C Ekezie, Charles I Ezema, Chioma Ikele, Vidhi Bhatt, Caitlin McArthur

**Affiliations:** Faculty of Health, School of Kinesiology and Health Science, York University, Toronto, Ontario, Canada; Emerging Researchers and Professionals in Ageing—African Network, Toronto, Canada; Department of Health Research Methods, Evidence and Impact, Faculty of Health Science, McMaster University, Hamilton, Canada; Emerging Researchers and Professionals in Ageing—African Network, Toronto, Canada; Emerging Researchers and Professionals in Ageing—African Network, Toronto, Canada; School of Physiotherapy, Dalhousie University, Halifax, Nova Scotia, Canada; Emerging Researchers and Professionals in Ageing—African Network, Toronto, Canada; Emerging Researchers and Professionals in Ageing—African Network, Toronto, Canada; Department of Medical Rehabilitation, Faculty of Health Science and Technology, University of Nigeria, Enugu Campus, Nigeria; Department of Medical Rehabilitation, Faculty of Health Science and Technology, University of Nigeria, Enugu Campus, Nigeria; Department of Medical Rehabilitation, Faculty of Health Science and Technology, University of Nigeria, Enugu Campus, Nigeria; Temerty Faculty of Medicine, University of Toronto, Toronto, Ontario, Canada; School of Physiotherapy, Dalhousie University, Halifax, Nova Scotia, Canada

**Keywords:** Aged, Associations, Movement, Sub-Saharan Africa

## Abstract

**Background and Objectives:**

Although the association between self-reported and capacity-based mobility outcomes is prominently researched, the pathways through which self-reported measures affect capacity-based measures remains poorly understood. Therefore, our study examines the association between self-reported and capacity-based mobility measures and explores which mobility determinants mediate the association in Nigerian community-dwelling older adults.

**Research Design and Methods:**

This cross-sectional study included 169 older adults [mean age (*SD*) = 67.7 (7.0)]. Capacity-based mobility outcomes included the Short Physical Performance Battery (SPPB), the 6-Minute Walk Test (6MWT), and the 10-Meter Walk Test (10mWT), whereas the self-reported mobility outcomes included the Lower Extremity Functional scale (LEFS), the Life Space Questionnaire (LSQ), and the Mänty Preclinical Mobility scale (inability to walk 2 km, 0.5 km, or climb a flight of stairs). Spearman’s correlations were conducted to examine the relationship between self-reported and capacity-based mobility measures, whereas structural equation modeling was used to determine the mediators.

**Results:**

The correlation between SPPB and LEFS (rho = 0.284) and 0.5 km (rho = −0.251) were fair, whereas the correlation between SPPB and inability to walk 2 km (rho = −0.244) and inability to climb a flight of stairs (rho = −0.190) were poor. Similarly, correlations between 6MWT and the LEFS (rho = 0.286), inability to walk 2 km (rho = −0.269), and 0.5 km (rho = −0.303) were fair. The 6WMT was poorly correlated with inability to climb one flight of stairs (rho = −0.233). The LSQ was not correlated with SPPB or 10mWT. Age was the only significant mediator, whereas the number of chronic conditions and cognitive status were not.

**Discussion and Implications:**

The correlation between self-reported and capacity-based mobility outcomes in older adults in Nigeria is lower than those in developed countries. Our analysis provides a foundation to explore mobility determinants that could be predictive mediators for mobility outcomes, making meaningful contributions to explaining mobility complexities.

**Translational Significance:** Culture plays a role in mobility assessment, specifically in self-reported measures. The association between self-reported and capacity-based mobility outcomes in Nigeria ranged from poor to fair, lower than those obtained in the world’s developed regions. Could culture play a role in this lower association? A qualitative study could explore this. In addition, age, but not chronic conditions and cognitive status, mediates this association, highlighting the continued importance of the predictive mediatory role on mobility outcomes.

Mobility is vital to healthy, active, successful, optimal, and positive aging as it has been noted as a precursor for many health outcomes among the older population (Kalu et al., 2022a). Conversely, mobility limitations have been associated with reduced socialization, increased risks of falls, hospitalization, and mortality among older adults (Freiberger et al., 2020). In addition, older adults with mobility limitations require additional help with instrumental and personal activities of daily living, such as running errands and bathing, resulting in additional healthcare costs (Hardy et al., 2011). The importance of mobility among older adults has been highlighted by Canadian and United States specialists in geriatric medicine, who previously launched the GERIATIC 5Ms framework (Schwartz et al., 2020), emphasizing the need to measure mobility in clinical and community practice.

Mobility is measured subjectively or objectively. Subjective measures of mobility, generally referred to as self-reported measures, represent the person’s perception of their mobility, including their behaviors, beliefs, attitudes, or intentions regarding mobility (Holbrook, 2008; Nielsen et al., 2016). Self-reported mobility measures are participant’s reported or researcher-administered mobility questions inquiring about the following, but not limited to the distance walked, number of times walked (seconds or minutes per week/month), number of days outdoors and self-reported ability to walk up and down a flight of stairs (10 steps) or several flights of stairs, walking a mile (1600-m) or half a mile (800-m) or a quarter-mile or a block (400-m) or across the room measuring life space. Other self-reported mobility measures include life space questionnaires, Rosow–Breslau Mobility Disability scale (Rosow & Breslau, 1966), and Late-Life Function and Disability Instrument (Jette et al., 2002), and self-reported ability to drive a car, driving parameters, such as duration and frequency and access to car and transportation. Although self-reported mobility measures provide information that captures meaningful mobility activities to older adults, they can either over or underestimate mobility (Fillekes et al., 2019) and are prone to recall and response bias leading to social desirability which limit capturing “real” or “close to real” mobility measures (Holbrook, 2008).

Objective measures are commonly used to increase precision and accuracy and validate self-reported measures. Historically, researchers have described all objective measures as performance-based, previously defined as assessments conducted in a standardized environment or controlled lab. However, with the advent of technology, such as accelerometers that measure real-life mobility, the World Health Organization, through the International Classification of Functioning, Disability, and Health, has distinguished capacity- and performance-based mobility measures (World Health Organization, 2005). Capacity indicates an individual’s highest possible level of functioning in a given domain at a particular moment and is often captured in a standardized environment or labs (World Health Organization, 2005). In contrast, performance-based measures are real-life assessments of what individuals can do in their environment (Giannouli et al., 2016; World Health Organization, 2005). Regardless, objective measures are believed to measure precise estimates combating the recall bias and social desirability associated with self-reported measures. However, objective measures are often time-consuming, require space, are expensive to conduct, and in most cases, require specialized skills to administer. Evidence has reported that older adults who are unwell or injured may temporarily lack the ability to complete the objective mobility measures, advocating for administering self-reported mobility measures to such individuals (Reuben et al., 2004). Although the use of both self-reported and objective mobility measures is recommended (Reuben et al., 2004), this might not be achieved because of practice (limited time), environment (space to conduct the test), and/or personal (older adults’ ability due to sickness) factors. Therefore, exploring the association between self-reported and objective mobility measures is needed to justify using one over the other, such as using self-reported measures over objective measures in situations where older adults might not be able to perform capacity-based measures because of previous mobility limitations, such as using a wheelchair or temporary weakness following a recent hospitalization.

Studies have compared self-reported and objective physical functioning measures in community-dwelling older adults (Baker et al., 2003; Peel et al., 2005; Sainio et al., 2006; Simonsick et al., 2001; Sydall et al., 2015). A systematic review including 17 articles conducted in developed countries found differing moderate to large relationships (correlations ranging from 0.60 to 0.86) between self-reported mobility measures, such as the Rosow–Breslau scale, and capacity-based mobility measures, such as gait speed and balance tests (Coman & Richardson, 2006). These differing correlations highlight the need for studies in different contexts, especially in developing countries with a population of predominantly different cultures and races. A scoping review identified that in general, Whites had faster gait speed, higher life space scores, or access to transportation and amenities promoting mobility than Blacks and Hispanics, and these differences could be due to the accumulated exposure to adverse health events and disadvantages that Blacks and Hispanics face across their life course (Kalu et al., 2023). Similarly, Whites have been found to overestimate their physical activity level compared to Blacks (Bazargan-Hejazi et al., 2017).

With this pattern of Black older adults under-reporting outcomes related to health, exploring the association between self-reported and capacity-based mobility measures among Black older adults in a developing country, such as Nigeria, is pertinent. As such, the first objective of this study was to examine the association between self-reported and capacity-based mobility measures among Black older adults in Nigeria. Among community-dwelling older adults in Nigeria, we hypothesized that although the Lower Extremity Functional Scale & Life Space Questionnaire (self-reported) would positively correlate with the capacity-based mobility measures [Short Performance Battery (SPPB), 6-Minute Walk Test (6MWT) and 10-Meter Walk Test (10mWT)], Mänty Preclinical Limitation Scale (self-reported) would negatively correlate with the capacity-based mobility measure **(H1).** We chose these mobility measures, including the SPPB, 6MWT, 10mWT, LSQ, and LEFS, based on their clinical relevance in Nigeria, as these are commonly used by therapists, ensuring the practical applicability of our findings, and they also have strong psychometric properties with prior validation in a population similar to Nigeria’s (Pinto-Carral et al., 2016; Tumuslime et al., 2014).

Furthermore, investigating mobility factors mediating the relationship between self-reported and capacity-based measures is pertinent because it will help explain the underlying mechanism and pathways through which self-reported affects depends on capacity-based measures, providing a deeper understanding of the relationship to better inform policies. Previous studies have reported a relationship between mobility factors and self-reported or capacity-based mobility measures. Using the United States InCHIANTI longitudinal study of 14 years of follow-up, Ferrucci et al. reported that an increase in age is associated with a decrease in performance-based walking speed, highlighting the influence of age on performance-based measures (Ferrucci et al., 2016). In a Nigerian population, Nwachukwu et al. (2023) reported that poor cognitive status, an increase in age, and several chronic conditions were associated with reduced gait speed and lower extremity strength. Similarly, self-reported mobility limitation increases with age (Kuspinar et al., 2020), as older adults tend to underestimate their functional limitations relative to younger people due to reduced expectations for self-performance. In addition, a relationship exists between self-reported mobility measures, chronic conditions, and cognitive status (Ndubuka et al., 2023; Nwachukwu et al., 2023). However, it is unclear if this relationship entails a causal relationship with self-reported measures regressing on age, chronic conditions, and cognitive status. Nonetheless, Webber et al.’s Conical Model of the Theoretical Framework for Mobility highlighted the interrelationships explaining mobility complexity, including, but not limited to, how mobility factors interact with each other to mediate or moderate mobility outcomes (Webber et al., 2010). Our previous work reported that living arrangement, defined as the number of individuals living in the same household, is a significant moderator (interacts with predictors to change the strength or direction of the relationship between predictors and outcomes) in both self-reported and capacity-based mobility in community-dwelling older adults (Nwachukwu et al., 2023). However, the extent to which these determinants may act as mediators (explain the relationship between two variables) to link self-reported mobility (as a predictor) to capacity-based mobility measures (as an outcome) is yet to be explored among community-dwelling older adults in the world’s developing regions. Therefore, the second objective of this study was to explore the mediating role of mobility determinants in the relationship between self-reported and objective-based measured mobility among community-dwelling older adults in Nigeria. Therefore, we hypothesized that the association between self-reported and capacity-based mobility measures would be partially mediated by age, number of chronic conditions and cognitive status of community-dwelling older adults in Nigeria **(H2)**.

## Method

### Study Design and Participants

This cross-sectional study recruited community-dwelling older adults using a multistage cluster sampling approach to identify communities and conveniently sample participants in each selected community. Participants were included if they were 60 years or older, able to ambulate with or without aid, follow instructions, and speak and understand English. We excluded participants that self-reported recent cardiovascular events (within 6 months), significant hearing or vision impairment, fractures (within 4 months), severe arthritis, vestibular disorders, and uncontrolled hypertension. Ethics approval was sought and approved from the Health Research Ethics Committee, University of Nigeria Teaching Hospital, Ituku Ozala-NHREC/05/01/2008B-FWA00002458-IR000002323. Only participants who provided written and oral consent were allowed to participate. A well-detailed description of the study sampling has been described elsewhere (Nwachukwu et al., 2023). We reported our study in accordance with the AGReMA (A Guideline for Reporting Mediation Analyses) statement (Supplementary Table 1; Lee et al., 2021).

### Sample Size Calculation

We followed Fritz & Mackinnon’s (2007) guideline for determining the sample size necessary to conduct mediational analysis using 0.8 statistical power. For a partial mediation of small variance (τʹ = 0.14), we estimated a sample size between 118 and 562 is needed for 0.8 power if the size of α and β variance is small (0.14) to moderate (0.39), building on existing literature that has explored factors mediating self-reported and performance-based reports of physical health among older adults (Varallo et al., 2022; Warner et al., 2012).

### Capacity-Based Measures

*The Short Physical Performance Battery (SPPB)* is a valid and reliable physical functioning tool that consists of three tests assessing older adults’ gait speed (4-Meter Walk Test), lower extremity physical performance (Chair Rise Test), and balance (Tests of Standing Balance; Guralnik et al., 1995). Participants completed a *4-meter walk,* and the time recorded was used to calculate their gait speed. The *Tests of Standing Balance* consist of side-by-side, semitandem, and tandem stand tests. Participants started by standing side-by-side with their feet apart, and if they could complete this in 10 s, they proceeded to semitandem, where the participants placed the heel of one foot to the side of the first toe of the other foot. If participants completed semitandem in 10 s, they proceeded to a tandem stand, where they placed the heel of one foot directly in front of the toes of the other foot. Finally, the participants were asked to perform the Chair Rise Test by standing up from a chair with their arms across their chest, and if possible, they were asked to stand up and sit down five times as quickly as possible. The time required to complete five sit-to-stand transitions was recorded, assessing participants’ lower extremity strength. Each gait speed, balance and lower extremity strength was rated on a scale of 0–4, with a summary score ranging from 0 to 12, and the highest score indicating better physical performance.

*The Six Minute Walk Test (6MWT)* measures the distance an individual can walk over 6 min on a hard or flat surface; initially, the 6MWT was developed to measure cardiopulmonary functioning and endurance (Lipkin et al., 1986). However, researchers have used the 6MWT as a proxy for physical functioning and it has been validated among community-dwelling adults (Harada et al., 1999; Lipkin et al., 1986). We followed the American Thoracic Society 2002 guideline for the 6MWT, where the walking course was 30 m long. We marked the turnaround points using an orange traffic cone. Participants were informed to walk as fast as possible for 6 min along the walking course; advised not to talk to anyone during the test; however, they were permitted to slow down, stop, and rest as necessary. After the 6MWT, participants rested for 1 h before coming to other capacity-based mobility measures (American Thoracic Society, 2002).

*The 10-meter Walk Test (10mWT)* is a validated and reliable tool measuring gait speed (Peters et al., 2013). Participants were asked to walk with or without aid at their preferred walking speed along a 10-meter walkway without any rest at the endpoint. Gait speed was calculated using the time required to cover the middle 4-meter of the walkway, allowing for acceleration and deceleration effects. Participants repeated the test twice, and the mean was used as the gait speed in this study.

### Self-Reported Measures

*The Lower Extremity Functional Scale (LEFS)* is a reliable questionnaire containing 20 questions about an individual’s ability to perform everyday tasks using their lower limbs (Binkley et al., 1999). Participants answered each of the 20 questions, for example, do you have any difficulty walking between rooms, squatting, getting in and out of a bath or a car, using extremely difficult/unable to perform (0), quite a bit difficulty (1), moderate difficulty (2), a little bit of difficulty (3), and no difficulty (4). The 20 questions are summed, and scores range from 0 to 80; the lower the score, the greater the disability. A systematic review has reported that among older adults, LEFS demonstrated excellent test–retest reliability of intraclass correlation coefficient ranging from 0.85 to 0.99 and Pearson correlation values of greater than 0.7, with excellent responsiveness score of high effect sizes of greater than 0.8 (Mehta et al., 2016). Items of LEFS have been noted to be moderately correlated (intra-assessor reliability of 0.7) among a sample of people living with HIV in Rwanda (Tumusiime et al., 2014), a similar setting for our study.

*The Mänty Preclinical Limitation scale* is a validated and reliable self-reported mobility measure assessing older adults’ mobility limitations (Mänty et al., 2007). Participants were asked to describe how difficult it was walking 2 km, 0.5 km, or climbing a flight of stairs using five options—without difficulty, some difficulty, a great deal of difficulty, manage only with help for another person or unable to manage even with the help of another person (Mänty et al., 2007). We grouped those without difficulty and some difficulty as having no mobility limitations, whereas participants that reported other categories were classified as having mobility limitations. Mobility limitations related to walking 2 km, 0.5 km, or climbing a flight of stairs were analyzed separately.

*The Life Space Questionnaire (LSQ)* is a moderately valid and reliable self-reported measure of participants’ life space (Stalvey et al., 1999; Ullrich et al., 2022),. The questionnaire consists of nine questions asking participants about the areas they have visited in the past three days (with or without walking aids). Such areas included other rooms in your home besides the rooms you sleep, outside your home such as, patio or parking lot, outside apartment building, outside neighborhood but within town or community, outside town or community, outside states and region and country. Participants answered yes or no to each of the nine questions, and we scored yes as one and no as zero, and scores ranged from 0 to 9, with the highest score indicating large life space and better mobility.

#### Mediators

Age, number of chronic conditions, and cognitive status were considered mediators for this study. Participants self-reported their age and chronic conditions, and research assistant counted the self-reported chronic conditions. Cognitive status was assessed using Montreal Cognitive Assessment (MoCA), a validated and reliable 30-point test measuring short-term, working memory, executive functioning, attention, and language (Nasreddine et al., 2005). The MoCA has been validated in several sub-Saharan African countries with similar sociocultural context to Nigeria (Beath et al., 2018; Masika et al., 2021).

### Data Collection

All measures were done in Wave 1 (April 2019 to June 2019) and Wave 2 (November 2020 to January 2021). In every wave, data was collected in a community hall. The SPPB (capacity-based measures), LEFS, and Mänty Preclinical Limitation scale (self-reported) were collected in both waves. Although the 6MWT (capacity-based) was collected in only Wave 1, LSQ and 10mWT were collected in Wave 2. Regardless of the wave, participants first responded to self-reported mobility measures, followed by objective measures (capacity-based). Participants rested in between capacity-based measures as needed. A registered physical therapist did all assessments.

### Data Analysis

Data were analyzed using STATA/SE (v17) with a *p*-value for significance set at < .05. The descriptive analysis was presented as means and standard deviations (for continuous variables) and frequency/percentages (for noncontinuous variables). The differences between Waves 1 and 2 in demographic variables and mobility measures were tested using a two-sided *t*-test for continuous variables and a chi-square test for noncontinuous variables. We calculated Cronbach’s alpha to evaluate the internal consistency of the MoCA, LEFS, and LSQ, as these tools have not been tested in the Nigerian population.

Mediation models were tested using self-reported mobility measures (LEFS and Mänty Preclinical Limitation scale domains; as a predictor) and capacity-based mobility measures (SPPB; as an outcome), age, number of chronic conditions, and MoCA scores as mediators, only in the combined waves. Mediation models were not conducted in the individual waves due to insufficient sample sizes. The mediating role of age, number of chronic conditions, and MoCA scores was examined compliant with guidelines from Baron and Kenny (1986). First, Spearman’s correlations were used to investigate the relationship between (a) SPPB (capacity-based) and the LEFS and Mänty Preclinical Limitation scale domains (self-reported); and (b) age and the LEFS and Mänty Preclinical Limitation scale domains (self-reported). Coefficients were interpreted as poor (<0.25), fair (0.25–0.49), moderate (0.50–0.74), and excellent (≥0.75) (Portney, 2007). Second, the predictors (self-reported measures) were not normally distributed, so a nonparametric approach (bootstrap) of structural equation modeling was used to test the mediation models. The bootstrapping approach is ideally used for small samples and allows us to use the confidence interval derived by the approach to infer the significant effects (direct, indirect, and total) without assuming a normal distribution. Effects were considered significant when the interval did not include zero (Preacher & Hayes, 2012). The path coefficients are the unstandardized beta (*B*) coefficients of the structural equation models and represent the magnitude and direction of the associations between variables included in the models.

## Results

In total, 169 older adults (82 for Wave 1 and 87 for Wave 2) met the criteria for participation in the study and had available data. Compared to participants in Wave 1, older adults in Wave 2 were younger, worked more skilled jobs, obtained greater educational qualifications, had higher incomes, exercised more frequently, had lower mean arterial blood pressures, had better cognitive capabilities, and were less likely to self-report mobility limitations. There were no differences in sex, marital status, comorbidities, body mass index, and living arrangements between Waves 1 and 2. Table 1 describes the two waves’ demographics and mobility measures and their differences. In the present sample, the Cronbach’s alpha of the MoCA was 0.9181. Similarly, the Cronbach’s alpha of the LSQ and the LEFS were 0.6602 and 0.9404, respectively.

**Table 1.** Demographic Characteristics of the Older Adults (*n* = 227)

Participant characteristics	Wave 1 (*n* = 90)	Wave 2 (*n* = 137)	Statistical test^e^
Age (years), mean (SD)	69.2 (8.1)	66.3(5.8)	*t* (167) = 2.68, *p* = .008
Sex (female), *n* (%)	49 (59.8%)	47(54.0%)	χ^2^ (1) = 0.57, *p* = .452
Occupation, *n* (%) ^a^			χ^2^ (2) = 9.33, *p* = .009
Skilled	7 (8.5%)	16 (18.4%)	
Semiskilled	5 (6.1%)	14 (16.1%)	
Unskilled	67 (81.7%)	53 (60.9%)	
Missing data	3 (3.7%)	4 (4.6%)	
Education, *n* (%)			χ^2^ (3) = 9.80, *p* = .020
No educational qualification	23 (28.0%)	25 (28.7%)	
Primary	44 (53.7%)	19 (21.8%)	
Secondary	9 (11.0%)	19 (21.8%)	
Tertiary	6 (7.3%)	14 (16.1%)	
Missing data	-	–	
Marital status, *n* (%)			χ^2^ (1) = 1.98, *p* = .160
Married	56 (68.3%)	52 (59.8%)	
Widowed/divorced/single	23 (28.0%)	34 (39.1%)	
Missing data	3 (3.7%)	1 (1.1%)	
Monthly household income, *n* (%)^b^			*t* (105) = −3.36, *p* = .001
Below poverty line	41 (50.0%)	30 (34.5%)	
Above poverty line	13 (15.9%)	23 (26.4%)	
Missing data	28 (34.1%)	34 (39.1%)	
Comorbidities, *n* (%)			*t* (166) = 0.16, *p* = .537
0–2 conditions	68 (82.9%)	75 (86.2%)	
>2 conditions	13 (15.9%)	12 (13.8%)	
Missing data	1 (1.2%)	–	
Frequency of exercise, *n* (%)			χ^2^ (3) = 92.37, *p* < .001
None	59 (72.0%)	4 (4.6%)	
Daily	9 (11.0%)	53 (60.9%)	
Weekly	3 (3.7%)	12 (13.8%)	
Other	4 (4.9%)	17 (19.5%)	
Missing data	7 (8.5%)	1 (1.1%)	
BMI, mean (*SD*)	25.4 (6.8)	26.4 (5.6)	*t* (166) = −1.03, *p* = .304
MAP mean (*SD*)	105.5 (20.3)	99.0 (13.4)	*t* (166) = 2.44, *p* = .016
MoCA score mean (*SD*)	16.2 (7.0)	22.4 (7.0)	*t* (165) = −5.67, *p* < .001
Living arrangement mean (*SD*)	4.8 (3.6)	4.6 (2.8)	*t* (165) = 0.31, *p* = .760
SPPB total score mean (*SD*)^c^	8.63 (3.32)	9.1 (1.9)	*t* (167) = 0.48, *p* = .632
LEFS total score mean (*SD*)^d^	54.7 (17.3)	65.0 (19.9)	*t* (167) = −3.60, *p* < .001
Manty 2 km walk, *n* (%)			χ^2^ (1) = 17.92, *p* < .001
No limitation	40 (48.8%)	70 (80.5%)	
Limitation	41 (50.0%)	17 (19.5%)	
Missing data	1 (1.2%)	–	
Manty 0.5 km walk, *n* (%)			χ^2^ (1) = 12.22, *p* < .001
No limitation	46 (56.1%)	71 (81.6%)	
Limitation	35 (42.7%)	16 (18.4%)	
Missing data	1 (1.2%)	–	
Manty stair climb, *n* (%)			χ^2^ (1) = 24.23, *p* < .001
No limitation	30 (36.6%)	65 (74.7%)	
Limitation	51 (62.2%)	22 (25.3%)	
Missing data	1 (1.2%)	–	

*Notes*: BMI = body mass index; LEFS = lower extremity functional scale; MAP = mean arterial pressure; MoCA = Montreal Cognitive Assessment scale; *SD* = standard deviation; SPPB = Short Physical Performance Battery; *t* = *t*-statistic; χ2 = chi-square statistic.

^a^Occupational classification: skilled = civil servants (retired and active), mechanical engineer, teacher, barrister; semiskilled = vulcanizer, tailor, carpenter, hotel supervisor, builder, welder, self-employed, business owner; unskilled = cleaner, farmer, trader, security officer, gateman, none.

^b^Poverty line was set at the adjusted $1.90/day.

^c^SPPB scores ranged from 0 to 12, with higher scores indicating higher physical performance. Each subset, including gait speed, balance, and chair rise test, scored 0–4, with 4 indicating the highest performance in each subset.

^d^LEFS scores ranged from 0 to 80 in which the lower the score, the greater the disability.

^e^Statistical tests comparing Waves 1 and 2.

### Correlation Analyses

#### Combined wave

In the combined wave, the Lower Extermity Function scale (LEFS) and Short Physical Performance Battery (SPPB) test had a ceiling effect; 43 (25.4%) and 18 (10.7%) older adults scored the highest score possible on the LEFS and SPPB, respectively. The mean (SD) scores were 60.0 (19.3) for the LEFS and 9.2 (2.2) for the SPPB. Fifty-eight (34.5%) and 51 (30.4%) older adults self-reported limitations in walking 2 km and 0.5 km, respectively. Seventy-three (43.4%) older adults self-reported limitations in climbing one flight of stairs.

The correlation between SPPB and self-reported measures varies: SPPB versus LEFS, inability to walk 2 km, and inability to walk 0.5 km were fair. Meanwhile, the correlation between SPPB and the inability to climb one flight of stairs was poor. See Table 2 for strength and direction. See Supplementary Table 2 for correlation matrix between all measures used in combined wave.

**Table 2.** Correlation Analyses Between the SPPB and Self-Reported Mobility Measures

Self-reported mobility measure	*N*	Spearman’s rho (95%CI)	*p*-Value
LEFS	169	0.284 (0.139 to 0.417)	0.0002
Manty 2 km walk	168	−0.244 (−0.382 to −0.097)	0.0014
Manty 0.5 km walk	168	−0.251 (−0.387 to −0.103)	0.0010
Manty stair climb	168	−0.190 (−0.332 to −0.040)	0.0136

*Notes*: CI = confidence interval; LEFS = lower extremity functional scale; SPPB = short physical performance battery.

#### Individual waves

In Wave 1, the correlations between the 6MWT and the LEFS (rho 0.286, 95% CI 0.066–0.479), self-reported limitations walking 2 km (rho = −0.269, 95%CI −0.466 to −0.046), and self-reported limitations walking 0.5 km (rho = −0.303, 95%CI −.495 to −.083) were all fair. The 6WMT was poorly correlated with self-reported limitations in climbing one flight of stairs (rho = −0.233, 95%CI −.435 to −.007; Supplementary Table 3). In Wave 2, the 10mWT was not significantly correlated with the LEFS, the LSQ, and self-reported limitations walking 2 km, 0.5 km, and climbing one flight of stairs. The SPPB was also not significantly correlated with the LSQ (Supplementary Table 4).

### Mediation Analyses

**Figure 1.**
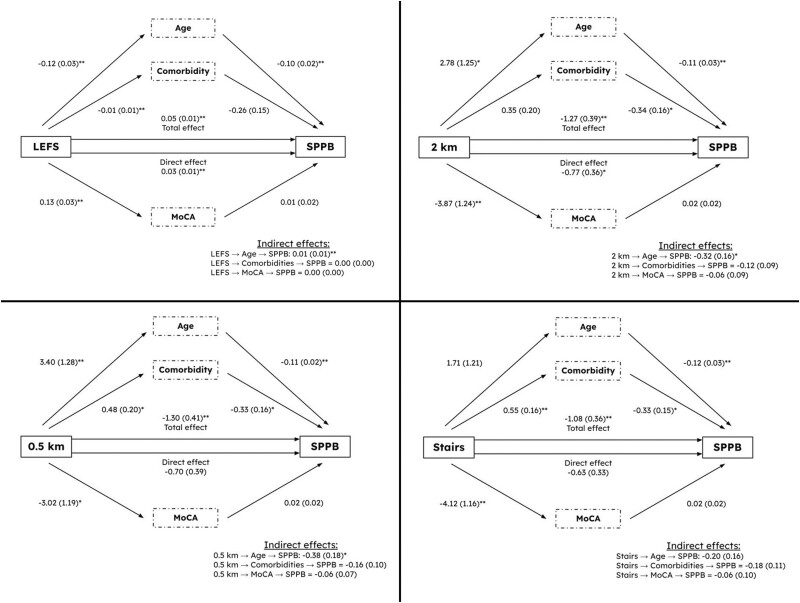
Mediation analysis between capacity-based mobility measure—short physical performance battery test (SPPB) versus self-reported mobility measures. (**a**) Lower extremity function scale (LEFS), (**b**) inability to walk 2 km, (**c**) inability to walk 0.5 km, and (**d**) inability to climb a stair. MoCA = Montreal Cognitive Assessment scale.

We tested how LEFS scores affected SPPB scores through three hypothesized mediators: age, number of comorbidities, and MoCA score. Only age was a significant mediator between (a) LEFS and SPPB scores (*p* = .007), with a poor fit model (χ^2^ = 10.3, *p* = .016; RMSEA = 0.120; CFI = 0.928; TLI = 0.760); (b) self-reported limitations walking 2 km and SPPB performance (*p* = .042), with a poor fit model (χ^2^ = 13.7, *p* = .003; RMSEA = 0.146; CFI = 0.848; TLI = 0.495); (c) self-reported limitations walking 0.5 km and SPPB performance (*p* = .033), with a poor fit model (χ^2^ = 14.6, *p* = .002; RMSEA = 0.152; CFI = 0.836; TLI = 0.453). No variable mediated the relationship between self-reported limitations climbing one flight of stairs and SPPB performance (Figure 1).

## Discussion

Although studies reporting the relationship between self-reported and capacity-based mobility outcomes do exist, their findings are equivocal (Baker et al., 2003; Peel et al., 2005; Sainio et al., 2006; Sydall et al., 2015), and most were from developed regions, including North America, Europe, or Oceanic regions (Coman & Richardson, 2006). Plausible reasons for the equivocal findings could be related to some underlying mechanism explaining the relationship. Nevertheless, studies that explore the underlying mechanism (mediators) between self-reported and capacity-based mobility outcomes are scarce. To the best of our knowledge, this study is the first to examine the relationship between several self-reported (LEFS, Mänty Preclinical Limitation Scale, LSQ) and capacity-based mobility (SPPB, 6MWT, 10mWT) outcomes in a developing context while exploring the mediating factors chosen from comprehensive mobility determinants (Kalu et al., 2022a, 2022b, 2023). We found the correlation between self-reported (LEFS and Mänty Preclinical Limitation scale) and capacity-based (SPPB and 6MWT) mobility measures ranged from poor to fair, in the expected direction. For instance, although LEFS was fairly and positively correlated with SPPB, self-reported limitation in climbing one flight of stairs (Mänty Preclinical Limitation scale) was poorly and negatively correlated with the SPPB. We found no significant correlation between 10mWT versus LSQ or SPPB versus LSQ. Although age mediates the association between self-reported and capacity-based mobility measures, the number of chronic conditions or cognitive status did not.

We could partially confirm the first hypothesis that self-reported mobility measures significantly correlate with capacity-based mobility measures. Some self-reported measures (LEFS, inability to walk 2 km, 0.5 km, and climbing one flight of stairs) significantly but fairly or poorly correlated with the SPPB and 6MWT, whereas another self-reported measure—the LSQ—was not correlated with 10mWT or SPPB performance. These mixed findings regarding the correlations between self-reported and capacity-based mobility align with the existing literature of mixed results (Baker et al., 2003; Bean et al., 2011; Coman & Richardson, 2006; Kuspinar et al., 2020; Neilsen et al., 2016; Peel et al., 2005; Sainio et al., 2006; Simonsick et al., 2001; Sydall et al., 2015). Moreso, these mixed findings contribute to or reiterate that the correlation’s strength and direction depend on how mobility is measured and the study population. Our finding that SPPB scores fairly correlate with LEFS scores agrees with Bean et al.’s (2011) study but disagrees with Nielsen et al.’s (2016) study. Our study and Bean et al. (2011) used SPPB that measured a comprehensive physical function of balance, speed and lower limb extremity strength among community-dwelling older adults. Neilsen et al.’s study used Time Up Go Test (TUG) that measures basic mobility and reflects a person’s ability to get up from a chair, walk 3 m, and turn around (only semidynamic balance; Shubert et al., 2006). The comprehensive balance assessment, including tandem, semitandem, and stand side-by-side (a component of the SPPB), may have reduced the correlation from moderate in Nielsen et al.’s study to fair in our study and Bean et al.’s (2011). In addition, Nielsen et al. (2016) used the Barthel-20 (Sainsbury et al., 2005) that captures activities beyond mobility to include the person’s level of independence in their performance of daily activities, such as grooming, bathing, eating, as their self-reported measures.

Surprisingly, despite life space being a commonly used, reliable and valid self-reported measure that captures how an individual move or travel across several spatial areas, including their bedroom, home, outside the home, neighborhood, town, and globally (Stalvey et al., 1999), it did not significantly correlate with any capacity-based measures (10mWT and SPPB) in our study. This finding disagrees with previous studies that have reported a significant association between life space scores and gait speed (Kuspinar et al., 2020) or SPPB scores (Ullrich et al., 2019). The differences in the findings between our study and these studies could be based on how life space was measured. Although we measured life space using the original version—answering yes or no to questions (for instance—have you been outside your home in the last 3 days), Kusipnar et al. (2020) & Ullrich et al. (2019) studies measured using a different modified version of living space that accounted for the frequency of movement, for example, 1–3 times a week or 4–6 times a week. Regardless, we believe that the lack of correlation between the life space measure and capacity-based in our study could be that the questions asked in the life-space mobility are not culturally adaptable to the Nigerian culture, highlighting the need for cultural adaptation of life-space measures in other cultures and developing regions, rather than the developed region. Besides, life space measures have been adapted for several older adult population including those living with cognitive impairment residing in a nursing home (Ullrich et al., 2022), further addressing the importance of cultural adaptation of life space measures in cultures different from the original version. We hoped that our findings would help clinicians to choose self-reported mobility measures for older adults who are not able to perform capacity-based measures. However, we cannot confidently recommend using self-reported mobility measures alone, as they only partly correlate with capacity-based mobility measure; underscoring the importance of using both when necessary (Reuben et al., 2004).

We hypothesized that age, number of chronic conditions, and cognitive status could partially mediate the association between self-reported and capacity-based mobility measures; however, age was the only partial mediator in our study—increasing age reduced the strength of the association. This finding, however, reiterates the importance of age in mobility assessment, enhancement and intervention among older adults. We proceeded with caution in interpreting this finding, as there are inherent problems with cross-sectional mediation analysis (Fairchild & McDaniel, 2017). Nevertheless, age is a well-established predictor of mobility outcomes (self-reported and capacity-based) and could explain why it partially mediates the association (Kalu et al., 2023; Nicolson et al., 2021). Moreso, age-related physiological (for example, reduced muscle mass or power), mechanical, such as joint articular degeneration due to “wear’ and “tear” and psych-cognitive changes unfold simultaneously with mobility decline among older adults could provide another plausible reason for age mediating role in the association between self-reported and capacity-based mobility outcomes (Freiberger et al., 2020). Regardless, this finding is purely exploratory, and a longitudinal mediation analysis with a large data set is required to confirm our study’s preliminary findings. After this, researchers can conclude the predictive mediatory role of age as a potential to select mobility assessment, intervention, or prevention in practice. This finding can help interventionists proactively recognize age as a mediating variable and create age-specific interventions that consider potential differences between self-reported and capacity-based measures for various age groups (Fairchild & McDaniel, 2017).

Inconsistent with Rhayun et al. (2023), our study found no mediating effect of cognition on the association between self-reported and capacity-based mobility. Rhayun et al. reported that cognition indirectly mediates the association between balance (not TUG) and physical components of quality of life. The mechanism through which this mediation occurs has been described as complex. However, scholars have speculated that activating the vestibular system that controls balance could enhance memory, an aspect of cognition (Hüfner et al., 2007; Smith et al., 2010). Notably, the capacity-based mobility measure used in our study is comprehensive, capturing balance, lower limb extremity, and gait speed whereas in Rhayun et al.’s study mobility was captured as balance; this could explain why cognition, which is theoretically linked to balance-related mobility, was not a mediator in our study because we measured more than balance via the SPPB.

Similarly, the number of chronic conditions did not mediate mobility outcomes in our study, as the number of chronic conditions has been known to be a better predictor of self-reported than capacity-based mobility measures (Bean et al., 2011). However, this finding may be related to the health status of our cohort, which had fewer chronic conditions than previous cohorts (Bean et al., 2011). Our finding highlighted that even though the number of chronic conditions strongly predict mobility outcomes, their mediating role is limited. Could the interaction between the number of chronic conditions and the types of chronic conditions better mediate the association between self-reported and capacity-based mobility measures than the number of chronic conditions? This question warrants further evaluation.

Although this study has provided insight into the correlation between self-reported and capacity-based mobility outcomes in a developing region as well as its mediator, there are limitations. Even though we followed a recommended approach to determining sample size for mediation analysis, our bootstrap approach may have elevated type 1 error (concluding there is an association when there is none) in our analysis (Fritz & MacKinnon, 2007). Although mediation analysis has been advocated to be conducted with a well-developed theoretical rationale, we based our choice of mediators to be tested based on empirical evidence from cross-sectional studies, and partially from a mobility theoretical framework (Webber et al., 2010), emphasizing that this study findings is exploratory highlighting the need for caution in interpretation of this study finding. Regardless, mediation analyses are better understood and explained when conducted with longitudinal data (Fairchild & McDaniel, 2017); however, our mediation analysis utilized cross-sectional data, limiting its causality interpretation. Nevertheless, our cross-sectional mediation analysis has provided a foundation to explore mobility determinants that could be predictive mediators for mobility outcomes, making meaningful contributions to further developing theories to explore the complexity associated with mobility, including its measures and use in practice. Another limitation relates to the reliability and validity of specific self-reported measures, such as the Manty Preclinical Disability Scale, which lacks validation in Nigeria or similar contexts. Sociocultural factors could influence respondents’ answers and scoring. For instance, most older adults in Nigeria might not be familiar with what distance equates to 2 km or 0.5 km. Cultural validation of the questionnaire using examples relative to 2 km or 0.5 km is recommended, in addition to assessing test–retest and interrater reliability in populations beyond those in the original psychometric testing conducted by Manty et al. (2007).

## Conclusion

The correlation between self-reported and capacity-based mobility measures ranged from poor to fair among community-dwelling older Nigerians. Although the LEFS was fairly and positively correlated with the SPPB, the inability to walk 2 km, 0.5 km, or climb a flight of stairs was poorly and negatively correlated with SPPB scores. We found no significant correlation between the LSQ and the 10mWT or SPPB. Only age mediates the association between self-report and capacity-based mobility measures, whereas chronic conditions or cognitive status did not. Interpretation of this study’s findings should be cautioned because of limitations related to the small sample size and the pitfalls of cross-sectional mediation analysis. Therefore, a longitudinal study with an extensive data set is needed to confirm the findings of this study.

## Supplementary Material

igae026_suppl_Supplementary_Tables

## Data Availability

The data supporting this study’s findings are not openly available due to reasons of sensitivity and are available from the corresponding author upon reasonable request.
