# Supplementary material for: Comparison of Self-Reported and Capacity-Based Measures of Mobility in Community-Dwelling Older Adults in Nigeria: The Mediating Role of Age, Cognitive Status, and Chronic Conditions
Source: Innov Aging. 2024 Feb 29;8(4):igae026. doi: 10.1093/geroni/igae026 (PMC11020311; doi:10.1093/geroni/igae026)
Supplement: igae026_suppl_Supplementary_Tables [file igae026_suppl_supplementary_tables.docx]

*Innovation in Aging* Supplementary Material: Michael E Kalu, Daniel Rayner, Ernest C Nwachuwku, Michael C Ibekaku, Miracle Ndukaku, Uduonu C Ekezie, Charles I Ezema, Chioma Ikele, Vidhi Bhatt, & Caitlin McArthur. Comparison of self-reported and capacity-based measures of mobility in community- dwelling older adults in Nigeria: The mediating role of age, cognitive status, and chronic conditions

**Supplemental Table 1.** Completed AGReMA statement.

| **Section/Topic** | **Item Number** | **Item Description** | **Reported on page No** |
| --- | --- | --- | --- |
| **Title and abstract** | | | |
| Title | 1 | Identify that the study uses mediation analysis | Page 1 |
| Abstract | 2 | Provide a structured summary of the objectives, methods, results, and conclusions specific to mediation analyses | Pages 1-2 |
| **Introduction** | | | |
| Background and rationale | 3 | Describe the study background and theoretical rationale for investigating the mechanisms of interest. Include supporting evidence or theoretical rationale for why the intervention or exposure might have a causal relationship with the proposed mediators. Include supporting evidence or theoretical rationale for why the mediators might have a causal relationship with the outcomes | Pages 3-8 |
| Objectives | 4 | State the objectives of the study specific to the mechanisms of interest. The objectives should specify whether the study aims to test or estimate the mechanistic effects | Pages 6-8 |
| **Methods** | | | |
| Study registration | 5 | If applicable, provide references to any protocols or study registrations specific to the mediation analysis, and highlight any deviations from the planned protocol | Not applicable |
| Study design and source of data | 6 | Specify the design of the original study that was used in mediation analyses and where the details can be accessed, supported by a reference. If applicable, describe study design features that are relevant to mediation analyses | Page 8 |
| Participants | 7 | Describe the target population, eligibility criteria specific to mediation analyses, study locations, and study dates (start of participant enrolment and end of follow-up) | Pages 8-14 |
| Sample Size | 8 | State whether a sample size calculation was conducted for mediation analyses. If so, explain how it was calculated | Page 9 |
| Effects of interest | 9 | Specify the effects of interest | Pages 12-13 |
| Assumed causal model | 10 | Include a graphic representation of the assumed causal model including the exposure, mediator, outcome, and possible confounders | Figure 1 |
| Causal assumptions | 11 | Specify assumptions about the causal model | Pages 6-8 |
| Measurement | 12 | Clearly describe the interventions or exposures, mediators, outcomes, confounders, and moderators that were used in the analyses. Specify how and when they were measured, the measurement properties, and whether blinded assessment was used | Pages 9-14 |
| Measurement levels | 13 | If relevant, describe the levels at which the exposure, mediator, and outcome were measured | Pages 9-14 |
| Statistical methods | 14 | Describe the statistical methods used to estimate the causal relationships of interest. This description should specify analytical strategies used to reduce confounding, model building procedures, justification for the inclusion or exclusion of possible interaction terms, modelling assumptions, and methods used to handle missing data. Provide a reference to the statistical software and package used | Pages 13-14 |
| Sensitivity analyses | 15 | Describe any sensitivity analyses that were used to explore causal or statistical assumptions and the influence of missing data | Not applicable |
| Ethical approval | 16 | Name the institutional research board or ethics committee that approved the study. Provide a description of participant informed consent or ethics committee waiver of informed consent | Page 8 |
| **Results** | | | |
| Participants | 17 | Describe baseline characteristics of participants included in mediation analyses. Report the total sample size and number of participants lost during follow-up or with missing data | Table 1 |
| Outcomes and estimates | 18 | Report point estimates and uncertainty estimates for the exposure-mediator and mediator-outcome relationships. If inference concerning the causal relationship of interest is considered feasible given the causal assumptions, report the point estimate and uncertainty estimate | Figure 1 |
| Sensitivity parameters | 19 | Report the results from any sensitivity analyses used to assess robustness of the causal or statistical assumptions, and the influence of missing data | Not applicable |
| **Discussion** | | | |
| Limitations | 20 | Discuss the limitations of the study including potential sources of bias | Pages 21-22 |
| Interpretation | 21 | Interpret the estimated effects considering the study’s magnitude and uncertainty, plausibility of the causal assumptions, limitations, generalizability of the findings, and results from relevant studies | Pages 16-21 |
| Implications | 22 | Discuss the implications of the overall results for clinical practice, policy, and science | Pages 19-20 |
| **Other information** | | | |
| Funding and role of sponsor | 23 | List all sources of funding or sponsorship for the mediation analysis and the role of the funders/sponsors in the conduct of the study, writing of the manuscript, and decision to submit for publication. | Not applicable |
| Conflicts of interest and financial disclosures | 24 | State any conflicts of interest and financial disclosures for all authors | Not applicable |
| Data and code | 25 | Authors are encouraged to provide a statement for sharing data and code for the mediation analysis | Not applicable |

**Supplemental Table 2.** Correlation matrix between all mobility measures and mediators in the combined wave (n = 168).

| **Variable** | **Age** | **Number of comorbidities** | **MoCA** | **SPPB** | **LEFS** | **Manty 2km walk** | **Manty 0.5km walk** |
| --- | --- | --- | --- | --- | --- | --- | --- |
| **Number of comorbidities** | -0.0133 | - |  |  |  |  |  |
| **MoCA** | -0.2372* | -0.1494 | - |  |  |  |  |
| **SPPB** | -0.3332* | -0.2092* | 0.2045* | - |  |  |  |
| **LEFS** | -0.2597* | -0.2534* | 0.3607* | 0.2840* | - |  |  |
| **Manty 2km walk** | 0.1587* | 0.1193 | -0.2437* | -0.2444* | -0.6196* | - |  |
| **Manty 0.5km walk** | 0.2088* | 0.1702* | -0.1790* | -0.2507* | -0.5852* | 0.8548* | - |
| **Manty stair climb** | 0.2674 | 0.2153* | -0.2696* | -0.1901* | -0.5858* | 0.5758* | 0.5704* |

**Note:** * p-value < 0.05

Hint: LEFS – Lower Extremity Functional Scale; MoCA – Montreal Cognitive Assessment; SPPB – Short Physical Performance Battery

**Supplemental Table 3.** Correlation analyses between all capacity-based and self-reported mobility measures in Wave 1.

| **Mobility measure comparison** | **N** | **Spearman’s rho (95%CI)** | **P-value** |
| --- | --- | --- | --- |
| *Capacity-based vs. self-reported* | | | |
| 6MWT vs. LEFS | 77 | 0.286 (0.066 to 0.479) | 0.0118 |
| 6MWT vs. Manty 2 km walk | 76 | -0.269 (-0.466 to -0.046) | 0.0188 |
| 6MWT vs. Manty 0.5 km walk | 76 | -0.303 (-0.495 to -0.083) | 0.0078 |
| 6MWT vs. Manty stair climb | 76 | -0.233 (-0.435 to -0.007) | 0.0433 |
| *Capacity-based vs. Capacity-based* | | | |
| 6MWT vs SPPB | 79 | 0.505 (0.320 to 0.654) | <0.0001 |

**Note:** No self-reported measures were exclusive to Wave 1. For comparisons between self-reported measures (i.e., LEFS vs. Manty 2 km walk) see Supplemental Table 1.

Hint: 6MWT – Six Minute Walk Test; LEFS – Lower Extremity Functional Scale; SPPB – Short Physical Performance Battery.

**Supplemental Table 4.** Correlation analyses between all capacity-based and self-reported mobility measures in Wave 2.

| **Mobility measure comparison** | **N** | **Spearman’s rho (95%CI)** | **P-value** |
| --- | --- | --- | --- |
| *Capacity-based vs. self-reported* | | | |
| 10mWT vs. LEFS | 87 | 0.096 (-0.117 to 0.301) | 0.3745 |
| 10mWT vs. Manty 2 km walk | 87 | -0.060 (-0.267 to 0.153) | 0.5806 |
| 10mWT vs. Manty 0.5 km walk | 87 | 0.015 (-0.196 to 0.225) | 0.8920 |
| 10mWT vs.Manty stair climb | 87 | 0.099 (-0.115 to 0.303) | 0.3640 |
| 10mWT vs. LSQ | 136 | 0.041 (-0.129 to 0.208) | 0.6388 |
| SPPB vs. LSQ | 136 | 0.116 (-0.053 to 0.279) | 0.1790 |
| *Capacity-based vs. Capacity-based* | | | |
| 10mWT vs SPPB | 137 | 0.444 (0.299 to 0.570) | <0.0001 |
| *Self-reported vs. Self-reported* | | | |
| LSQ vs LEFS | 87 | 0.221 (0.010 to 0.412) | 0.0209 |
| LSQ vs. Manty 2 km walk | 87 | -0.121 (-0.324 to 0.092) | 0.4219 |
| LSQ vs. Manty 0.5 km walk | 87 | -0.102 (-0.306 to 0.111) | 0.3407 |
| LSQ vs. Manty stair climb | 87 | -0.054 (-0.262 to 0.158) | 0.5175 |

Hint: 10mWT – 10-meter Walk Test; LEFS – Lower Extremity Functional Scale; LSQ – Life Space Questionnaire; SPPB – Short Physical Performance Battery.
